# Interpretable machine learning for detecting symptomatic patients with carotid atherosclerosis on computed tomography angiography: a retrospective diagnostic study

**DOI:** 10.1186/s12880-025-02113-1

**Published:** 2025-12-22

**Authors:** Yulu Yang, Jianyong Wei, Xiaoer Wei, Xinyu Song, Zhiwen Yang, Zheng Sun, Chao Zheng, Shundong Hu, Li Zhuo, Yueqi Zhu, Yuehua Li

**Affiliations:** 1 Institute of Diagnostic and Interventional Radiology, Shanghai Sixth People’s Hospital Affiliated to Shanghai Jiao Tong University School of Medicine, No. 600 Yishan Roa, Shanghai, 200233 China; 2 School of Health Science and Engineering, University of Shanghai for Science and Technology, Shanghai, 200093 China; 3 Clinical Research Center, Shanghai Sixth People’s Hospital Affiliated to Shanghai Jiao Tong University School of Medicine, No. 600 Yishan Road, Shanghai, 200233 China; 4 ShuKun Technology Co., Ltd., Beichen Century Center, Chaoyang District, Beijing, 300401 China

**Keywords:** Carotid atherosclerosis, Perivascular adipose tissue, Computed tomography angiography, Radiomics, Machine learning

## Abstract

**Background:**

This study aimed to develop a machine learning (ML) model based on radiomics features of carotid plaques and perivascular adipose tissue (PVAT) on computed tomography angiography (CTA) to detect symptomatic carotid atherosclerosis.

**Methods:**

This retrospective study included patients with extracranial carotid atherosclerotic plaques who underwent CTA between January 2022 and January 2024. Patients were divided into symptomatic and asymptomatic groups based on the occurrence of cerebrovascular events within two weeks prior to the CTA examination. Five ML models were constructed to identify symptomatic patients: clinical, PVAT radiomics, plaque radiomics, PVAT and plaque radiomics, and combined model. The most robust model was selected for Shapley Additive Explanations (SHAP) analysis to visualize the prediction process.

**Results:**

The study cohort consisted of 229 patients (127 symptomatic; 102 asymptomatic). The Random Forest models demonstrated the best performance in detecting symptomatic patients. In the test cohort, the area under the curve (AUC) of the combined model (0.86; 95% confidence interval [CI]: 0.74–0.95) was significantly higher than that of the clinical model (AUC: 0.67, 95% CI: 0.50–0.81; *p* = 0.03), but similar to that of the PVAT and plaque radiomics model (AUC: 0.82, 95% CI: 0.70–0.93; *p* = 0.65). SHAP analysis of the combined model identified carotid plaque texture features and cholesterol levels as key factors in detecting symptomatic patients.

**Conclusions:**

Integrating radiomics of carotid plaques and PVAT with clinical data enhances the detection of symptomatic patients.

**Supplementary Information:**

The online version contains supplementary material available at 10.1186/s12880-025-02113-1.

## Introduction

Stroke is the second-leading cause of death and the third-leading cause of disability worldwide, making it a significant public health issue [1]. Extracranial arterial atherosclerosis accounts for approximately 15%–20% of all cases of ischemic stroke [2, 3]. Atherosclerosis is a complex pathological process characterized by abnormal lipid deposition in the arterial walls and infiltration of inflammatory cells [4]. Inflammation plays a pivotal role in the destabilization of atherosclerotic plaques, with perivascular adipose tissue (PVAT) acting as a critical site for the release of inflammatory mediators [5]. Although inflammatory serum biomarkers can reflect the presence of inflammation, they cannot indicate the exact areas of vascular inflammation [6, 7].

Computed tomography angiography (CTA) is a widely used efficient imaging modality for evaluating carotid atherosclerosis [8]. In patients with cerebrovascular ischemic symptoms, increased perivascular fat density (PFD) on CTA images of the ipsilateral carotid artery can serve as an imaging biomarker for vascular inflammation [9]. Moreover, carotid PFD is significantly associated with vulnerable plaque characteristics, including intraplaque hemorrhage, thin and/or ruptured fibrous caps, and American Heart Association type VI [10, 11]. However, carotid PVAT segmentation is challenging, necessitating manual methods to delineate regions of interest on two-dimensional slices and analyze Hounsfield units (HU) [9, 12–14]. Furthermore, the pathological- and physiological-level interactions of carotid plaques with the surrounding PVAT have often been overlooked. Inflammation originating from the plaque may extend outward into the adjacent perivascular fat, leading to compositional changes and increased fat density [15].

Radiomics technology enables automatic extraction of quantitative features, transforming digital medical images into high-dimensional data [16–18].It was hypothesized that specific radiomics features extracted from CTA images of carotid plaques and their corresponding PVAT may help detect symptomatic patients. Furthermore, since traditional machine learning (ML) methods often lack interpretability, Shapley Additive Explanations (SHAP) can serve as an effective solution to the interpretability problem and provide clinically actionable insights [19].

This study aimed to automatically segment carotid plaques and PVAT on CTA images and developed ML models combining the radiomics features of carotid plaques and PVAT for detecting symptomatic patients. SHAP was employed to visualize the model inference process, and the diagnostic values of traditional CTA models, radiomics models, and fusion models were compared.

## Materials and methods

### Study population

This retrospective, diagnostic study was approved by the Institutional Review Board of our institution (No. 2024-KY-097[K]) and informed consent was not required. A total of 345 consecutive patients with extracranial carotid atherosclerotic plaques who underwent both head and neck CTA and MRI within a two-week interval were enrolled at our hospital between January 2020 and January 2024. Patients were excluded if they had any one of the following conditions: symptoms caused by cardioembolic thrombi determined by the Trial of Org 10,172 in Acute Stroke Treatment criteria [20], posterior circulation occlusion; concomitant carotid artery diseases (such as dissection, aneurysm), intracranial diseases, or carotid stenosis resulting from radiation therapy; previous carotid surgery (such as stent placement, endarterectomy, or thrombectomy); poor-quality CTA images; and incomplete clinical information.

**Fig. 1:**
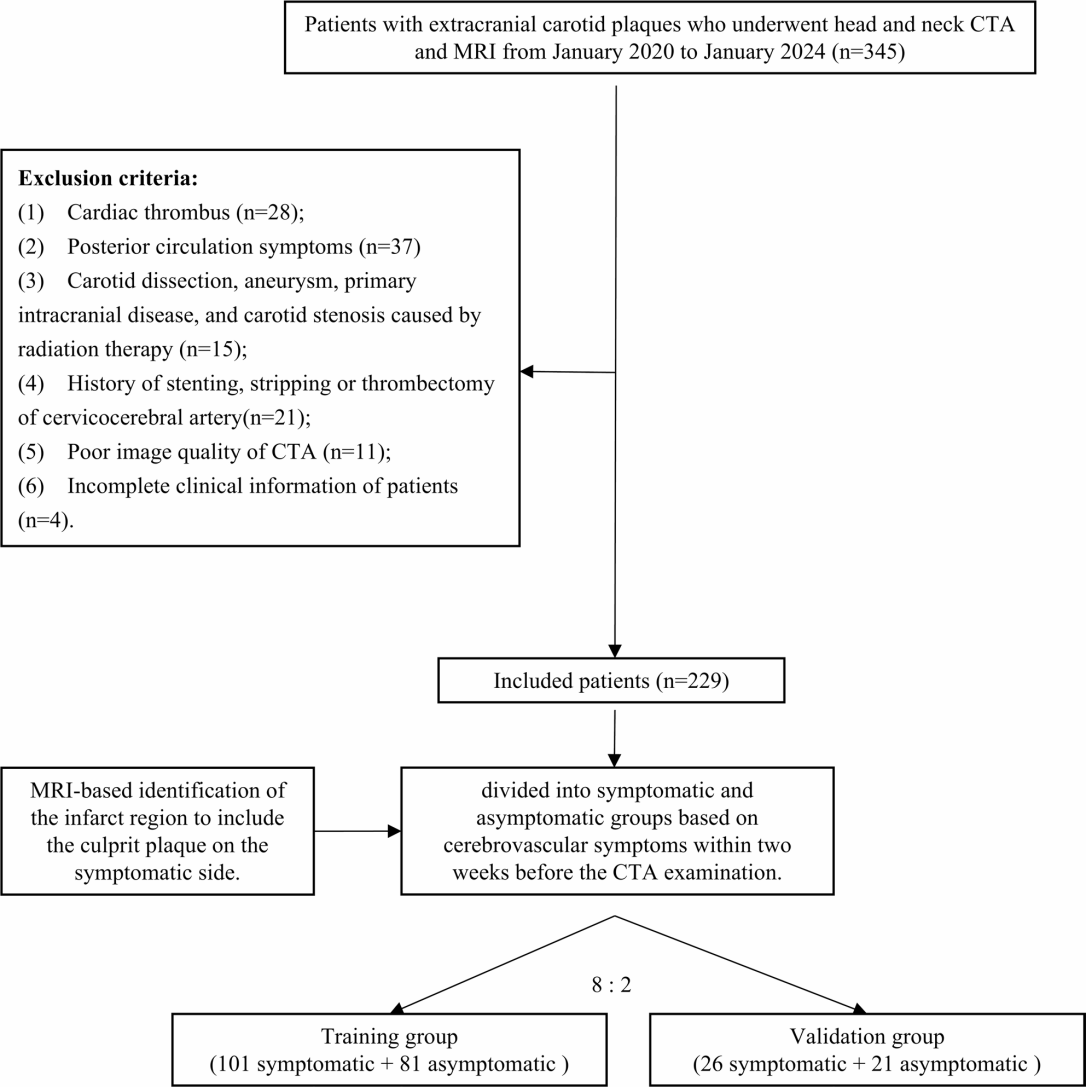
Flowchart of the study population. CTA, computed tomography angiography

A total of 229 patients were finally included and randomly divided into training (*n* = 182) and test (*n* = 47) cohorts (Fig. 1). The following clinical information was recorded: age, sex, body mass index, history of antiplatelet medication use (aspirin or clopidogrel), statin use (atorvastatin), history of diabetes, hypertension, smoking, hyperlipidemia, and coronary heart disease.

### Patient classification

All patients were divided into symptomatic and asymptomatic groups according to whether they had clinical symptoms within 2 weeks before the MRI examination and/or whether their head MRI showed ischemic stroke [21]. The clinical symptoms included acute ischemic stroke or transient ischemic attack affecting the anterior circulation [21]. Acute ischemic stroke was defined as permanent neurological dysfunction caused by focal cerebral or retinal ischemia lasting for more than 24 h [22]. Transient ischemic attack was defined as a transient neurological dysfunction caused by focal cerebral or retinal ischemia lasting less than 24 h, presenting with symptoms such as hemiparesis, speech impairment, or monocular blindness [22]. For patients with bilateral carotid artery atherosclerotic plaques, only the culprit plaque on the symptomatic side—identified via MRI-based localization of the infarct region within the territory supplied by the internal carotid artery—was included in the analysis [23]. Asymptomatic patients were defined as those without recent or remote cerebrovascular symptoms at the time of examination [24]. For these patients, only the side of the atherosclerotic carotid artery with more severe stenosis was included.

### CTA scanning parameters

CTA scans were performed using systems from four different manufacturers (Brilliance ICT, Philips; Somatom Sensation, Siemens; UIH; and GE Healthcare); the specific CT scanner models and parameters detailed in Table S1. Patients were positioned in the supine position with the head first. The scan range extended from the aortic arch to the top of the skull. A 50-mL injection of iodinated contrast agent was administered at a rate of 4.0–5.0 mL/s. A delayed scan was performed, triggered 3–4 s after reaching the attenuation threshold of 100 HU at the level of the aortic bifurcation.

### Segmentation of plaque and PVAT images

**Fig. 2:**
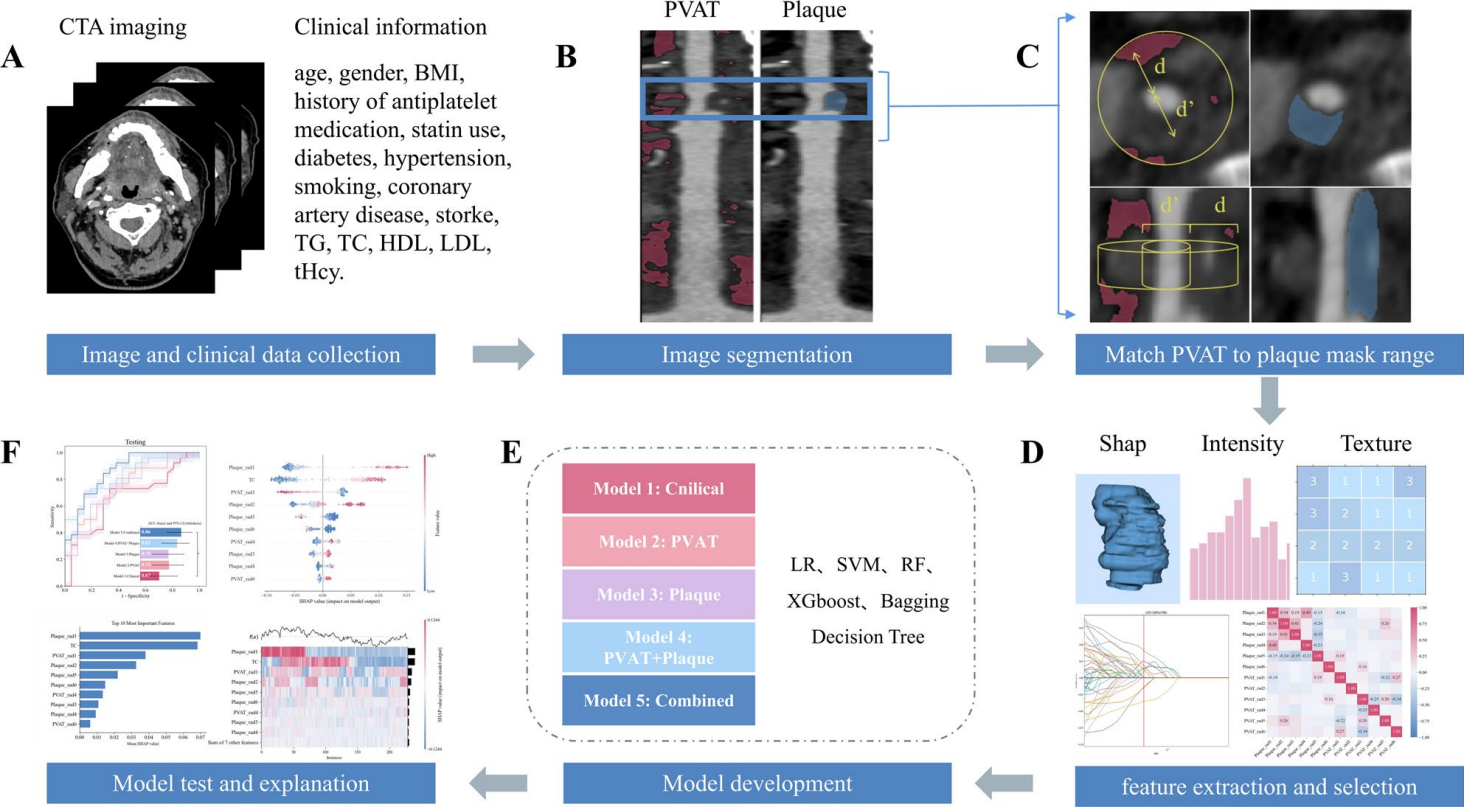
Flowchart of the study design. (**A**) CT image acquisition. (**B**) Segmentation of carotid artery plaques and surrounding adipose tissue. (**C**) Distribution of the perivascular adipose tissue (PVAT) region determined by the plaque boundaries. The red area represents adipose tissue with attenuation values between − 190 HU and − 30 HU. The PVAT width (d) represents the extent of the perivascular adipose tissue, while d’ corresponds to the diameter of the carotid artery lumen. The blue area indicates the corresponding plaque mask. (**D**) Extraction and selection of radiomics features. (**E**) Development of five models. (**F**) Results of the models on the test set and model visualization. CTA, CT angiography; PVAT, perivascular adipose tissue; BMI, body mass index; TC, total cholesterol; TG, triglycerides; HDL, high-density lipoprotein; LDL, low-density lipoprotein; tHcy, serum homocysteine

All images underwent anonymization. Data labeling was performed on the original axial images using ITK-SNAP software [25]. Preliminary segmentation of carotid vessels and plaques was performed using a developed deep learning (DL)-based segmentation model previously established by the author team (detailed in Fig. 2 and Figure S1) [26, 27]. Subsequently, to ensure annotation accuracy, two radiologists (both with more than 10 years of experience) blinded to the symptomatic status of the patients independently adjusted and supplemented the pre-annotated results with window width/level settings of 850/300 to obtain the final plaque annotations. They also assessed the degree of stenosis on the original CTA images on the basis of the North American Symptomatic Carotid Endarterectomy Trial criteria, classifying the stenosis as mild (< 50%), moderate (50%–69%), and severe (70%–99%) [28]. Disagreements regarding the final annotations and stenosis evaluations between the radiologists were arbitrated and reviewed by an expert (a senior radiologist with more than 15 years of experience).

After generating the straightened rendering images for plaques and PVAT segmentation, the upper and lower boundaries of the plaque mask were aligned with the segmented PVAT mask region. The PVAT region was defined as the area determined by the plaque boundaries, where the distribution of adipose tissue was characterized by a CT attenuation value ranging from − 190 HU to -30 HU [15]. Subsequently, the average, maximum, and minimum carotid artery PFD values were calculated on the basis of the matched PVAT mask, as illustrated in Fig. 2(c).

### Radiomics feature extraction and selection

Before radiomics feature extraction, all CT images were resampled to an isotropic voxel size of 1 mm × 1 mm × 1 mm, with a fixed bin width of 25 HU. Laplacian of Gaussian filters (kernel sizes: 0.5, 1, 1.5, and 2) and wavelet transformations were performed to enhance feature extraction. Radiomics features were extracted from the plaque and PVAT regions using PyRadiomics [20, 29]. In total, 1070 features were initially extracted, encompassing first-order statistical, shape-based, and texture features (including GLCM, GLRLM, GLSZM, GLDM, and NGTDM classes), as well as higher-order features derived from Laplacian of Gaussian and wavelet transformations. Radiomic features were extracted independently from the plaque and PVAT masks, and feature selection was performed separately for each region within the training cohort. First, z-score normalization was applied to standardize the extracted data. Second, the Wilcoxon–Mann–Whitney test was used to identify features with significant differences (*p* < 0.05). Subsequently, the least absolute shrinkage and selection operator method combined with 10-fold cross-validation was employed to further refine the selection of significant features. Additionally, the Pearson correlation coefficient was calculated to assess multicollinearity among features, with a correlation coefficient |r| >0.9 was considered indicative of high collinearity. In such cases, the feature with the smaller p-value was retained.

### Machine learning models development

A dataset consisting of 16 variables was used to develop the ML models (Table S2). These variables included traditional clinical features showing statistical significance (*p* < 0.05) in multivariate analysis, the degree of carotid artery stenosis, and radiomics features of carotid plaques and PVAT. The symptomatic status was treated as a binary classification outcome, with the aim of identifying symptomatic plaques. A 10-fold cross-validation strategy was used to train the models on the training cohort, and model performance was subsequently evaluated on the test set.

The overall workflow of the study is illustrated in Fig. 2. Several ML algorithms were applied, including extreme gradient boosting, bagging decision tree, random forest (RF), support vector machine and logistic regression (LR). A clinical model (Model 1) was developed using traditional clinical features that showed statistical significance (*p* < 0.05) in multivariate analysis. Radiomics models were constructed on the basis of PVAT (Model 2) and plaques (Model 3). A combined radiomics model (Model 4) was built by combining the radiomics features of plaques and PVAT. Finally, a combined model (Model 5) was developed by incorporating both traditional clinical features and the radiomics features.

### Model effectiveness

To ensure the stability and generalizability of the model, a 10-fold cross-validation method was applied during both training and performance evaluation. Model performance was assessed by generating receiver operating characteristic curves and calculating the area under the curve (AUC), sensitivity, specificity, and accuracy. The optimal classification threshold was determined using the Youden’s index, and evaluation metrics, including accuracy, sensitivity, and specificity, were calculated. A 95% confidence interval (CI) was estimated using 1,000 bootstrap samples to further enhance the robustness of the results.

## Importance of variables

The importance of features was ranked on the basis of their mean absolute impact across the entire dataset, as visualized using SHAP in the robust combined model. Additionally, two representative cases of correct predictions (symptomatic and asymptomatic) from the test cohort were presented to illustrate the individual contributions of each feature to the final prediction outcome.

### Statistical analysis

All statistical analyses were performed using SPSS (version 23.0, IBM Corp.), Python (version 3.11.6, Python Software Foundation), and MedCalc (version 18.2.1, MedCalc Software Ltd.). The sample size was calculated with the area under the receiver operating characteristic curve using PASS (detailed in Appendix E1). Categorical variables were expressed as frequencies and percentages, while continuous variables were presented as mean ± standard deviation based on normality. The χ² test was used for comparisons of categorical variables. The normality of continuous variables was assessed using the Shapiro–Wilk test, and normally distributed variables were compared using the *t* test. Variables showing statistical significance (*p* < 0.05) in univariate analysis were further analyzed using bidirectional stepwise LR. The diagnostic performance of the models was compared using the DeLong method, with *p* < 0.05 considered statistically significant.

## Results

### Patient characteristics

A total of 229 patients were enrolled in this study, including 127 symptomatic and 102 asymptomatic patients (mean age: 68.80 ± 8.71 years; 198 men). The demographic characteristics stratified by symptomatic status are summarized in Table 1 (see Additional file 1: Table 1 for details), with details of the training and test cohorts provided in Table S3. Univariate analysis revealed significant associations between symptomatic status and the minimum and mean PFD values (*p* < 0.05). Other significant factors included sex, smoking history, coronary artery disease history, statin use, total cholesterol (TC) levels, and low-density lipoprotein levels (*p* < 0.05). In multivariate LR analysis, symptomatic patients were more likely to be male (91.34% vs. 80.39%; *p* = 0.01), had higher TC levels (4.60 ± 1.39 vs. 3.95 ± 1.07, *p* < 0.01), higher mean PFD values (-56.23 ± 8.84 vs. -61.01 ± 10.47, *p* < 0.01), and were less likely to use statins (2.36% vs. 14.71%, *p* = 0.03) than asymptomatic patients.

**Table 1:** Patient characteristics in the symptomatic and asymptomatic subgroups

Characteristics	Total (*n* = 229)	Symptomatic (*n* = 127)	Asymptomatic (*n* = 102)	Statistic	Univariate *p* value	Multivariate	
						OR (95% CI)	*p* value
Age, (Mean ± SD, years)	68.80 ± 8.71	68.63 ± 9.88	69.02 ± 7.02	t = 0.35	0.73		
Gender, (male, n, %)	198 (86.46)	116 (91.34)	82 (80.39)	χ² = 5.79	0.02	3.32 (1.29 ~ 8.56)	0.01
BMI (Mean ± SD, kg/m2)	25.42 ± 20.01	23.70 ± 3.48	27.51 ± 29.53	t = 1.25	0.21		
Diabetes, n (%)	72 (31.44)	35 (27.56)	37 (36.27)	χ² = 1.99	0.16		
Hypertension, n (%)	165 (72.05)	92 (72.44)	73 (71.57)	χ² = 0.02	0.88		
Smoking, n (%)	58 (25.33)	40 (31.50)	18 (17.65)	χ² = 5.74	0.02	1.77 (0.83 ~ 3.75)	0.14
Coronary artery disease, n (%)	30 (13.10)	9 (7.09)	21 (20.59)	χ² = 9.06	0.003	0.58 (0.22 ~ 1.53)	0.27
Antiplatelet use, n (%)	12 (5.24)	4 (3.15)	8 (7.84)	χ² = 2.51	0.11		
Statin use, n (%)	18 (7.86)	3 (2.36)	15 (14.71)	χ² = 11.90	< 0.001	0.19 (0.04 ~ 0.82)	0.03
Antihypertension use, n (%)	69 (30.13)	37 (29.13)	32 (31.37)	χ² = 0.13	0.71		
Antidiabetic use, n (%)	98 (42.79)	57 (44.88)	41 (40.20)	χ² = 0.51	0.48		
History of stroke, n (%)	66 (28.82)	32 (25.20)	34 (33.33)	χ² = 1.83	0.17		
TC (Mean ± SD, mmol/L)	4.31 ± 1.30	4.60 ± 1.39	3.95 ± 1.07	t = -4.00	< 0.001	0.19 (0.04 ~ 0.82)	0.003
TG (Mean ± SD, mmol/L)	1.39 ± 0.79	1.40 ± 0.66	1.37 ± 0.93	t = -0.34	0.73		
LDL (Mean ± SD, mmol/L)	2.66 ± 1.41	2.89 ± 1.08	2.38 ± 1.70	t = -2.71	0.007	1.03 (0.74 ~ 1.45)	0.85
THcy (Mean ± SD, µmol/L)	14.37 ± 6.71	14.88 ± 5.80	13.50 ± 8.04	t = -1.36	0.18		
HDL (Mean ± SD, mmol/L)	1.09 ± 0.29	1.08 ± 0.32	1.09 ± 0.25	t = 0.10	0.92		
Min PFD (Mean ± SD, HU)	-122.59 ± 27.97	-117.54 ± 27.42	-128.88 ± 27.48	t = -3.11	0.002	1.01 (0.99 ~ 1.02)	0.56
Mean PFD (Mean ± SD, HU)	-58.36 ± 9.87	-56.23 ± 8.84	-61.01 ± 10.47	t = -3.75	< 0.001	1.87 (1.41 ~ 2.49)	< 0.001
Stenosis, n (%)				χ² = 1.88	0.39		
< 50	20 (8.73)	6 (5.88)	14 (11.02)				
50 ~ 69	26 (11.35)	12 (11.76)	14 (11.02)				
70 ~ 99	183 (79.91)	84 (82.35)	99 (77.95)				
Manufacturer, n (%)							
GE	50 (21.83)	22 (17.32)	28 (27.45)				
Philips	60 (26.20)	26 (20.47)	34 (33.33)				
SIEMENS	75 (32.75)	37 (29.13)	38 (37.25)				
UIH	44 (19.21)	42 (33.07)	2 (1.96)				

Note. The analyses were performed using χ² test and Student’s *t* test. Categorical variables are shown as frequency and percentage; continuous variables are shown as mean ± standard deviation (SD). BMI, body mass index; TC, total cholesterol; TG, triglycerides; HDL, high-density lipoprotein; LDL, low-density lipoprotein; tHcy, serum homocysteine; PFD, perivascular fat density; OR, odds ratio; CI, confidence interval

### Feature selection

A total of 1,070 quantitative features were extracted from the segmented images of plaques and PVAT (Table S4). Using the Wilcoxon–Mann–Whitney test, 254 and 342 plaque- and PVAT-related features, respectively, were retained. With a least absolute shrinkage and selection operator coefficient threshold of 0.015, six texture features were ultimately selected for plaques, while two first-order features and four texture features were selected for PVAT (Figure S2, Table S2).

### Performance of the models

**Fig. 3:**
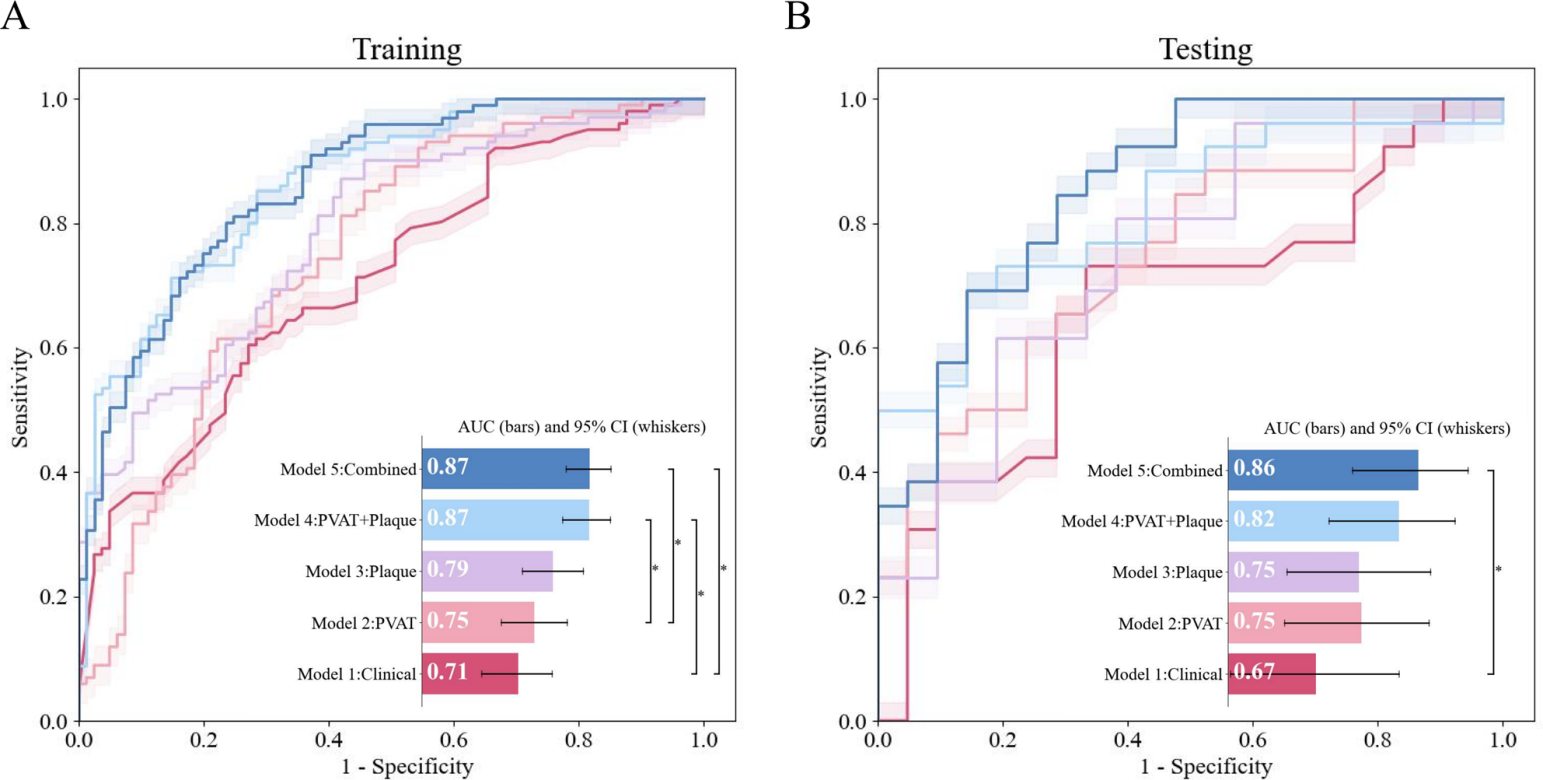
Evaluation of the performance results of the five models. Receiver operating characteristic (ROC) area under the curve (AUC) values for diagnosing symptomatic patients in the training (**A**) and test (**B**) cohorts. The median area under the curve is represented by horizontal bars, and the 95% confidence intervals (CIs) are indicated by whiskers. Comparisons between models are indicated by vertical whiskers annotated with asterisks to indicate statistical significance. PVAT, perivascular adipose tissue

Five ML algorithms, namely, LR, RF, support vector machine, extreme gradient boosting, and bagging decision tree, were employed to build and evaluate the five different models. The test set results, including the AUC, specificity, accuracy, and sensitivity for each model, are summarized in Table 2 (see Additional file 1: Table 2 for details; Table S5 for the training cohort). In both training and test datasets, the combined model achieved AUC values exceeding 0.85 and 0.82, respectively. No significant differences were observed between the RF model and the other four ML models (*p* = 0.21–0.85). However, in evaluations using comprehensive performance metrics, the RF-based model demonstrated superior stability across all datasets. Therefore, the RF algorithm was selected to build the optimal combined model (AUC = 0.86; 95% CI: 0.74–0.95; Fig. 3).

**Table 2:** TThe performance of different models in the testing cohort (*n* = 47)

Model	Machine Learning algorithm	Cutoff value	AUC (95% CI)	Specificity (%, 95% CI)	Sensitivity (%, 95% CI)	Accuracy (%, 95% CI)
Model 1: Clinical	SVM	0.57	0.62 (0.46, 0.77)	52.38 (31.02, 73.74)	5.85 (34.68, 73.01)	53.19 (38.93, 67.46)
	RF	0.56	0.67 (0.50, 0.81)	71.43 (52.11, 90.75)	53.85 (34.68, 73.01)	61.70 (47.80, 75.60)
	Bagging_Desicion_Tree	0.56	0.70 (0.55, 0.84)	52.38 (31.02, 73.74)	76.92 (60.73, 93.12)	65.96 (52.41, 79.50)
	XGBoost	0.54	0.69 (0.54, 0.83)	80.95 (64.16, 97.75)	57.69 (38.70, 76.68)	68.09 (54.76, 81.41)
	LR	0.51	0.60 (0.42, 0.76)	52.38 (31.02, 73.74)	57.69 (38.70, 76.68)	55.32 (41.11, 69.53)
Model 2: PVAT	SVM	0.55	0.73 (0.51, 0.83)	66.67 (46.50, 86.83)	69.23 (51.49, 86.97)	68.09 (54.76, 81.41)
	RF	0.58	0.75 (0.61, 0.87)	61.90 (41.13, 82.68)	69.23 (51.49, 86.97)	65.96 (52.41, 79.50)
	Bagging_Desicion_Tree	0.60	0.76 (0.60, 0.88)	90.48 (77.92, 100.00)	53.85 (34.68, 73.01)	70.21 (57.14, 83.29)
	XGBoost	0.51	0.76 (0.60, 0.89)	71.43 (52.11, 90.75)	69.23 (51.49, 86.97)	70.21 (57.14, 83.29)
	LR	0.43	0.71 (0.55, 0.86)	47.62 (26.26, 68.98)	92.31 (82.06, 100.00)	72.34 (59.55, 85.13)
Model 3: Plaque	SVM	0.53	0.76 (0.59, 0.90)	61.90 (41.13, 82.68)	92.31 (82.06, 100.00)	78.72 (67.02, 90.42)
	RF	0.55	0.75 (0.60, 0.83)	61.90 (41.13, 82.68)	80.77 (65.62, 95.92	72.34 (59.55, 85.13)
	Bagging_Desicion_Tree	0.55	0.76 (0.70, 0.86)	62.84 (43.36, 83.49)	76.92 (60.73, 93.12)	70.21 (57.14, 83.29)
	XGBoost	0.57	0.77 (0.61, 0.89)	71.43 (52.11, 90.75)	76.92 (60.73, 93.12)	74.47 (62.00, 86.93)
	LR	0.49	0.77 (0.62, 0.89)	61.90 (41.13, 82.68)	69.23 (51.49, 86.97)	65.96 (52.41, 79.50)
Model 4: PVAT + Plaque	SVM	0.52	0.81 (0.68, 0.92)	57.14 (35.98, 78.31)	80.77 (65.62, 95.92)	70.21 (57.14, 83.29)
	RF	0.58	0.82 (0.70, 0.93)	38.10 (17.32, 58.87)	96.15 (88.76, 100.00)	70.21 (57.14, 83.29)
	Bagging_Desicion_Tree	0.57	0.83 (0.70, 0.93)	76.19 (57.97, 94.41)	73.08 (56.03, 90.13)	74.47 (62.00, 86.93)
	XGBoost	0.50	0.82 (0.69, 0.93)	61.90 (41.13, 82.68)	76.92 (60.73, 93.12)	70.21 (57.14, 83.29)
	LR	0.47	0.81 (0.68, 0.92)	57.14 (35.98, 78.31)	84.62 (70.75, 98.48)	72.34 (59.55, 85.13)
Model 5: Combined	SVM	0.56	0.82 (0.68, 0.93)	61.90 (41.13, 82.68)	65.38 (47.10, 83.67)	63.83 (50.09, 77.57)
	RF	0.56	0.86 (0.74, 0.95)	71.43 (52.11, 90.75)	85.26 (71.18, 98.81)	78.72 (67.02, 90.42)
	Bagging_Desicion_Tree	0.54	0.84 (0.71, 0.93)	61.90 (41.13, 82.68)	84.62 (70.75, 98.48)	74.47 (62.00, 86.93)
	XGBoost	0.50	0.85 (0.73, 0.95)	85.71 (70.75, 100.00)	73.08 (56.03, 90.13)	78.72 (67.02, 90.42)
	LR	0.49	0.84 (0.72, 0.94)	71.43 (52.11, 90.75)	73.08 (56.03, 90.13)	72.34 (59.55, 85.13)

Note. PVAT, perivascular adipose tissue; SVM, support vector machines; RF, random forests; XGBoost, extreme gradient boosting; LR, logistic regression; AUC, area under the curve; CI, confidence interval

In the test dataset, Model 1 had an AUC of 0.67 (95% CI: 0.50–0.81), while Model 2 and Model 3 showed comparable performance with AUCs of 0.75 (95% CI: 0.61–0.87, *p* = 0.41) and 0.75 (95% CI: 0.60–0.83, *p* = 0.48), respectively. Model 4 achieved an AUC of 0.82 (95% CI: 0.70–0.93) with an overall specificity of 96.15%. Finally, Model 5 achieved the highest AUC of 0.86 (95% CI: 0.74–0.95). This model demonstrated specificity, sensitivity, and accuracy of 71.43%, 85.26%, and 78.72%, respectively. Its performance was significantly superior to that of Model 1 (*p* = 0.03) but comparable to that of Model 4 (AUC = 0.82; 95% CI: 0.70–0.93; *p* = 0.64).

### Random forest-based analysis of important variables for symptomatic plaques

**Fig. 4:**
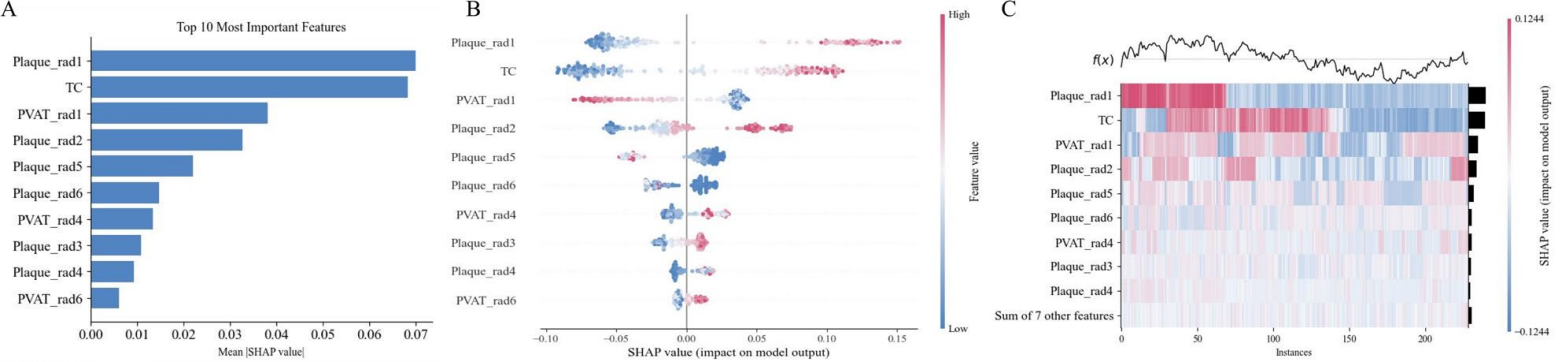
Visualization of the combined model using Shapley Additive Explanations (SHAP). (**A**) A bar chart summarizes features by their average SHAP values, with higher values indicating greater influence on the model. (**B**) The SHAP beeswarm plot shows each feature’s positive or negative influence on predicted probabilities in pink and blue. (**C**) The SHAP heatmap illustrates the influence of important features on final predicted probabilities. SHAP, Shapley Additive Explanations; TC, total cholesterol

To clarify the influence of variables on the identification of symptomatic plaques, the SHAP method was applied to the RF model. The SHAP summary bar plot (Fig. 4A) ranks the top 10 features by importance, with the corresponding abbreviations provided in Table S2. Additionally, a SHAP value plot (Fig. 4B) illustrated the effects of these features. The ranking of features along the y-axis indicates their importance to the model, with pink and blue dots representing positive and negative effects, respectively, on model outputs. Notably, higher values of plaque radiomics features, such as glrlm_ShortRunHighGrayLevelEmphasis (Plaque_rad1) and glszm_LowGrayLevelZoneEmphasis (Plaque_rad2), as well as elevated cholesterol levels, were associated with an increased likelihood of symptomatic plaques. Conversely, lower values of PVAT radiomics features, such as original_firstorder_Range (PVAT_rad1), were negatively associated with symptomatic plaques. The SHAP heatmap (Fig. 4C) further visualizes the direction and magnitude of these features’ impacts across all model cases.

**Fig. 5:**
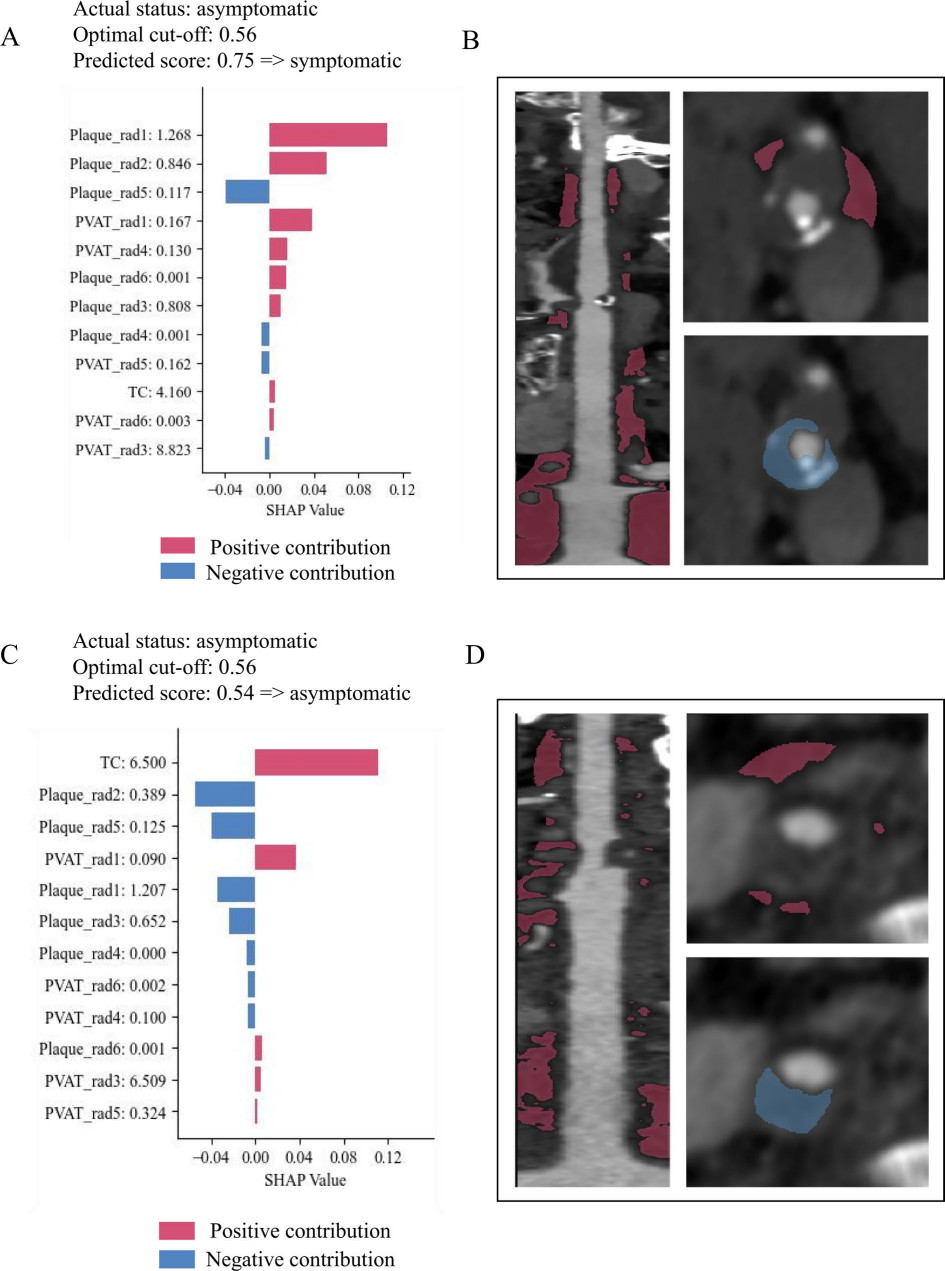
Examples of correctly diagnosed symptomatic and asymptomatic cases. (**A**-**C**) The individual contributions of each feature to the probability of detecting symptomatic status in the random forest model. Features are ranked in descending order of impact, with blue and pink bars representing negative and positive contributions, respectively. (**B**-**D**) Computed tomography angiography images showing a symptomatic carotid plaque in a man in his 60s and an asymptomatic carotid plaque in another man in his 60s

Patient-level SHAP visualizations were employed to interpret individual predictions. Figure 5 presents two representative cases correctly classified by the RF model. Figure 5A presents the findings for a male patient with a TC level of 4.16 mmol/L. The predicted probability for this patient was 0.75, exceeding the optimal cutoff value (0.56), leading to the classification of this patient as symptomatic. Conversely, Fig. 5C presents the findings for a male patient with a predicted probability of 0.54, which was below the cutoff value, leading to the classification of the patient as asymptomatic.

## Discussion

In this study, carotid artery plaques and PVAT were automatically segmented from CTA images, and carotid PFD was measured. By combining clinical features, a comprehensive model was developed and five commonly used ML algorithms were compared for the detection of symptomatic patients.The results demonstrated that the proposed model outperformed traditional radiological methods (AUC = 0.86 vs. 0.67, *p* = 0.03). Additionally, SHAP was used to visualize the diagnostic process at both overall and individual levels. These findings indicate that PVAT and plaque radiomics features are complementary and can help detect symptomatic patients.

Previous studies have primarily focused on the relationship between carotid PVAT and symptomatic or vulnerable plaque features. Baradaran et al. reported that increased carotid PFD on CTA is associated with a higher risk of cerebrovascular symptoms [9, 10]. Subsequent works by Luo et al. and Beşler et al. further confirmed its prognostic value in predicting symptomatic and ischemic events [30, 31]. However, these studies relied on manually placed two-dimensional regions of interest on axial slices, which are subject to operator variability and limited in capturing the spatial heterogeneity of perivascular inflammation. PFD also correlates significantly with plaque features, such as American Heart Association type VI and intraplaque hemorrhage [13]. Nie et al. achieved an AUC of 0.83 (95% CI: 0.69–0.98) for a bagging decision tree model based on carotid PVAT radiomics features [32]. Similarly, Wang et al. reported an AUC of 0.82 (95% CI: 0.76–0.88) using DL with carotid plaque radiomics features extracted from super-resolution 3D images [33]. Nevertheless, these models analyzed PVAT and plaque features separately, without accounting for their biological interplay [34]. It was believed that combining PVAT and plaque features can yield additional diagnostic value for identifying symptomatic patients. Concurrently, our model combining PVAT and plaque radiomic features (Model 4) demonstrated AUC and sensitivity values of 0.82 and 85.26%, respectively, in the test set.

Arterial tortuosity and branching, which are often influenced by surrounding tissues, present challenges in plaque and PVAT segmentation. Zhai et al. improved plaque detection and classification by first segmenting the carotid artery [35]. To address similar challenges, straightened carotid artery renderings were generated based on centerline extraction, and PVAT segmentation was performed to avoid deformation at arterial bifurcations. The PVAT regions were identified using defined threshold ranges. For plaque segmentation, a DL model was applied to the original axial CTA images, and the pre-annotated results were manually refined to improve accuracy. The DL-based model for automatic segmentation of carotid plaques and PVAT significantly reduced operator-induced variability and enhanced result consistency and reliability. Furthermore, PFD values were calculated from the segmented PVAT regions, demonstrating significant differences between symptomatic and asymptomatic groups (*p* = 0.03). This finding aligns with the results reported by Baradaran et al. However, the PFD values in their study were lower, possibly due to the 6-month threshold for symptomatic classification, which may reflect plaques in a more stable, less inflamed phase [36].

Advances in ML interpretability have addressed the “black-box” limitations associated with traditional models. SHAP allows visualization of individual feature contributions to predictions, enhancing model reliability and clinical acceptance. In the combined model, all top 10 contributing features were plaque radiomics features, particularly the texture features glszm_LowGrayLevelZoneEmphasis and glrlm_ShortRunHighGrayLevelEmphasis, which reflect low gray-level zones and short high-intensity regions, respectively. These features are associated with calcified plaques and vulnerable plaque characteristics, such as lipid-rich necrotic cores and intraplaque hemorrhage [24, 37]. The PVAT radiomics feature original_firstorder_Range, reflecting the intensity range (difference between maximum and minimum pixel values), also significantly influenced the model. SHAP visualization indicated that symptomatic patients had lower values of this feature. It was observed that the maximum PFD value for all patients was − 30 HU, indicating an increase in the minimum PVAT value among symptomatic patients. Since PVAT attenuation is a biomarker of inflammatory activity, this finding highlights the elevated inflammatory response in symptomatic patients [10, 38]. In addition, the results further confirm the diagnostic potential of increased carotid PFD values in identifying symptomatic patients.

This study had several limitations. First, it was a single-center, retrospective study with a small sample size, which may have introduced selection bias. Second, it did not include histopathological analyses to validate the inflammatory changes within plaques and PVAT. Third, the present study did not include a direct comparison between the proposed combined radiomics model and models built solely upon more established, traditional quantitative plaque assessment methods derived from CTA, such as total plaque volume, volumes of specific components (e.g., calcified plaque, low-attenuation plaque representing lipid-rich necrotic core), or plaque burden. While numerous studies have validated the utility of these traditional metrics (representative examples provided in Supplementary Table S6), incorporating them as a direct comparative benchmark was beyond the scope of this initial investigation focused on the synergy of plaque and PVAT radiomics. Future studies incorporating these traditional quantitative benchmarks, alongside larger sample sizes and longer follow-up periods, are required to fully elucidate the relative contributions and potential complementarity of advanced radiomics and established volumetric metrics, and to further validate these findings.

## Conclusion

In conclusion, the combination of plaque and PVAT radiomics features from CTA images demonstrated higher diagnostic accuracy and reliability than traditional CTA models in identifying symptomatic patients. Further research is warranted to explore the generalizability of this model and its potential clinical applications.

## Supplementary Information

Below is the link to the electronic supplementary material.

Supplementary Material 1

## Data Availability

The data that support the findings of this study are not publicly available due to privacy or ethical restrictions but are available on request from the corresponding author.

## Abbreviations

AUC: Area under the curve
CI: Confidence interval
CTA: Computed tomography angiography
DL: Deep learning
HU: Hounsfield units
LR: Logistic regression
ML: Machine learning
PFD: Perivascular fat density
PVAT: Perivascular adipose tissue
RF: Random forest
SHAP: Shapley Additive Explanations
TC: Total cholesterol
