# Supplementary material for: Interpretable machine learning for detecting symptomatic patients with carotid atherosclerosis on computed tomography angiography: a retrospective diagnostic study
Source: BMC Med Imaging. 2025 Dec 22;25:521. doi: 10.1186/s12880-025-02113-1 (PMC12751269; doi:10.1186/s12880-025-02113-1)
Supplement: Supplementary file 1 — Supplementary Material 1 [file 12880_2025_2113_MOESM1_ESM.docx]

**Appendix E1. Sample sizes calculation.** Our study employed a diagnostic approach, utilizing the PASS software (Version 21.0.3) to determine sample sizes. The sample size was calculated using the Area under the receiver operating characteristic curve (AUC). The smallest AUC was set as 0.85 according to the purpose of the study. Then, the Type I error α was set as 0.05, and the Type II error β was set as 0.1. The ratio of positive (symptomatic) to negative (asymptomatic) was approximately 2:3 [1]. The results analyzed by PASS showed that the smallest sample size required for model development was 115. When considering of 20% dropout rate, the required sample size was 145. Our study finally included 229 patients, which was align with the requirement.

**Table S1.** CT Scanning Parameters

| Scanner | Tube voltage (kv) | Tube current (mAs) | Detector collimation  (mm) | Fov (mm) | Pitch | Slice interval  (mm) | Slice thickness(mm) |
| --- | --- | --- | --- | --- | --- | --- | --- |
| Brilliance ICT, Philips | 100 | auto | 80 | 345×345 | 0.725 | 0.45 | 0.9 |
| Somatom Sensation, Siemens | 150 | auto | 58 | 250×250 | 0.7 | 0.5 | 1 |
| UIH | 100 | auto | 80 | 230×230 | 0.864 | 0.5 | 1 |
| GE MEDICAL SYSTEMSCT | 100-120 | auto | 40 | 250×250 | 0.984 | 0.625 | 0.625 |

**Table S2**. Description of variables used in machine learning.

| **Model 5: Combined**  (There are a total of 16 variables, including 4 traditional features and 6 radiomics features each for PVAT and plaque.) | **Model** | | **Name** | **Description** |
| --- | --- | --- | --- | --- |
|  | **Model 1: Clinical** | | TC | Total cholesterol level of the patient (mmol/L) |
|  |  |  | Stonesis | Degree of stenosis (%); <50%: 0; 50–69%: 1; 70–99%: 2 |
|  |  |  | Sex | Gender of the patient (male: 1; female: 0) |
|  |  |  | Statin use | Whether the patient has used statins in the past two weeks (true: 1; false: 0) |
|  | **Model 4: PVAT + Plaque** | **Model 2: PVAT** | **PVAT radiomics features** | |
|  |  |  | PVAT_rad1: original_firstorder_Range | The difference between the maximum and minimum gray values in the original image, measuring the overall variation in gray values. |
|  |  |  | PVAT_rad2: original_glszm_SizeZoneNonUniformity | The non-uniformity of region size calculated from the Gray Level Size Zone Matrix (GLSZM), reflecting the distribution of regions with the same gray values. |
|  |  |  | PVAT_rad3: log_sigma_0.5mm_glrlm_LongRunLowGrayLevelEmphasis | Importance of long-run low gray-level regions calculated from the Gray Level Run Length Matrix (GLRLM), with Gaussian smoothing (σ = 0.5 mm) and log transformation applied. |
|  |  |  | PVAT_rad4: log_sigma_1.5mm_glszm_SizeZoneNonUniformityNormalized | Normalized non-uniformity of region size calculated from the Gray Level Size Zone Matrix (GLSZM), with Gaussian smoothing (σ = 1.5 mm) and log transformation applied. |
|  |  |  | PVAT_rad5: wavelet-HHL_glszm_SmallAreaLowGrayLevelEmphasis | Significance of small low gray-level regions, using a combination of wavelet transformation (HHL sub-band) and the Gray Level Size Zone Matrix (GLSZM). |
|  |  |  | PVAT_rad6: wavelet-HHH_firstorder_90Percentile | Analysis of the 90th percentile in the distribution of gray values, using a combination of wavelet transformation (HHH sub-band) and first-order statistical features. |
|  |  | **Model 3: Plaque** | **Plaque radiomics features** | |
|  |  |  | Plaque_rad1: log_sigma_0.5mm_glrlm_ShortRunHighGrayLevelEmphasis | Importance of short-run high gray-level regions calculated from the Gray Level Run Length Matrix (GLRLM), with Gaussian smoothing (σ = 0.5 mm) and log transformation applied. |
|  |  |  | Plaque_rad2: log_sigma_2mm_glszm_LowGrayLevelZoneEmphasis | Importance of low gray-level zone size calculated from the Gray Level Size Zone Matrix (GLSZM), with Gaussian smoothing (σ = 0.5 mm) and log transformation applied. |
|  |  |  | Plaque_rad3: wavelet-LHL_glszm_LowGrayLevelZoneEmphasis | Quantification of the size of low gray-level regions, using a combination of wavelet transformation (LHL sub-band) and the Gray Level Size Zone Matrix (GLSZM). |
|  |  |  | Plaque_rad4: wavelet-HHH_glszm_ZonePercentage | Calculation of the distribution of same-gray-level regions in terms of percentage, using a combination of wavelet transformation (HHH sub-band) and the Gray Level Size Zone Matrix (GLSZM). |
|  |  |  | Plaque_rad5: wavelet-LLL_glszm_SmallAreaLowGrayLevelEmphasis | Significance of small regions with low gray levels, using a combination of wavelet transformation (LLL sub-band) and the Gray Level Size Zone Matrix (GLSZM). |
|  |  |  | Plaque_rad6: wavelet-LLL_ngtdm_Strength | Quantification of the strength of gray-level variations, using a combination of wavelet transformation (LLL sub-band) and the Neighborhood Gray-Tone Difference Matrix (NGTDM) to analyze image texture features. |

**Table S3**. Patient characteristics in the training and testing cohorts.

| Variables | Total (n = 229) | Training (n = 182) | Testing (n = 47) | Statistic | Univariate  *p* value |
| --- | --- | --- | --- | --- | --- |
| Age, (Mean ± SD, years) | 68.80 ± 8.71 | 68.92 ± 8.38 | 68.36 ± 9.95 | t = -0.39 | 0.70 |
| Gender, (male, n, %) | 198 (86.46) | 158 (86.81) | 40 (85.11) | χ² = 0.09 | 0.76 |
| BMI (Mean ± SD, kg/m2) | 25.42 ± 20.01 | 25.78 ± 22.60 | 24.12 ± 3.09 | t = -0.45 | 0.65 |
| Diabetes, n (%) | 72 (31.44) | 59 (32.42) | 13 (27.66) | χ² = 0.39 | 0.53 |
| Hypertension, n (%) | 165 (72.05) | 131 (71.98) | 34 (72.34) | χ² = 0.00 | 0.96 |
| Smoking, n (%) | 58 (25.33) | 41 (22.53) | 17 (36.17) | χ² = 3.68 | 0.06 |
| Coronary artery disease, n (%) | 30 (13.10) | 21 (11.54) | 9 (19.15) | χ² = 1.90 | 0.17 |
| Antiplatelet use, n (%) | 12 (5.24) | 8 (4.40) | 4 (8.51) | χ² = 0.58 | 0.45 |
| Statin use, n (%) | 18 (7.86) | 12 (6.59) | 6 (12.77) | χ² = 1.21 | 0.27 |
| Antihypertension use, n (%) | 69 (30.13) | 53 (29.12) | 16 (34.04) | χ² = 0.43 | 0.51 |
| Antidiabetic use, n (%) | 98 (42.79) | 81 (44.51) | 17 (36.17) | χ² = 1.06 | 0.30 |
| History of stroke, n (%) | 66 (28.82) | 52 (28.57) | 14 (29.79) | χ² = 0.03 | 0.87 |
| TC (Mean ± SD, mmol/L) | 4.31 ± 1.30 | 4.30 ± 1.34 | 4.37 ± 1.13 | t = 0.35 | 0.73 |
| TG (Mean ± SD, mmol/L) | 1.39 ± 0.79 | 1.39 ± 0.83 | 1.38 ± 0.64 | t = -0.10 | 0.92 |
| LDL (Mean ± SD, mmol/L) | 2.66 ± 1.41 | 2.67 ± 1.51 | 2.61 ± 0.95 | t = -0.26 | 0.80 |
| THcy (Mean ± SD, μmol/L) | 14.37 ± 6.71 | 14.35 ± 6.84 | 14.47 ± 6.25 | t = 0.10 | 0.92 |
| HDL (Mean ± SD, mmol/L) | 1.92 ± 12.41 | 1.10 ± 0.29 | 5.07 ± 27.27 | t = 1.94 | 0.05 |
| Min PFD (Mean ± SD, HU) | -122.59 ± 27.97 | -123.78 ± 27.96 | -117.98 ± 27.79 | t = 1.27 | 0.21 |
| Mean PFD (Mean ± SD, HU) | -58.36 ± 9.87 | -58.52 ± 9.32 | -57.75 ± 11.85 | t = 0.47 | 0.64 |
| Stenosis, n (%) |  |  |  | χ² = 1.53 | 0.465 |
| <50 | 20 (8.73) | 18 (9.89) | 2 (4.26) |  |  |
| 50~69 | 26 (11.35) | 20 (10.99) | 6 (12.77) |  |  |
| 70~99 | 183 (79.91) | 144 (79.12) | 39 (82.98) |  |  |
| Manufacturer, n (%) |  |  |  |  |  |
| GE | 50 (21.83) | 41 (22.53) | 9 (19.15) |  |  |
| Philips | 60 (26.20) | 45 (24.73) | 15 (31.91) |  |  |
| SIEMENS | 75 (32.75) | 57 (31.32) | 18 (38.30) |  |  |
| UIH | 44 (19.21) | 39 (21.43) | 5 (10.64) |  |  |

Note. χ² Chi-squared test; t Student t-test; Categorical variables shown with frequency and percentage; continuous variables shown with mean ± standard deviation (SD); BMI, body mass index; TC, total cholesterol; TG, triglycerides; HDL, high density lipoprotein; LDL, low density lipoprotein; tHcy, serum homocysteine; PFD, perivascular fat density.; OR, odds ratio; CI, confidence interval.

**Table S4.** List of the radiomic features extracted on CTA (*n* =1070).

| **ImageType** |
| --- |
| Original |
| LoG: sigma: [0.5, 1.0, 1.5, 2.0] |
| Wavelet |
| **featureClass** |
| **Shape (n=17)**  VoxelVolume; MeshVolume; SurfaceArea; SurfaceVolumeRatio; Compactness1; Compactness2; Sphericity; SphericalDisproportion; Maximum3DDiameter; Maximum2DDiameterSlice; Maximum2DDiameterColumn; Maximum2DDiameterRow; MajorAxisLength; MinorAxisLength; LeastAxisLength; Elongation; Flatness |
| **Firstorder (18)**  Energy; TotalEnergy; Entropy; Minimum; 10Percentile; 90Percentile; Maximum; Mean; Median; InterquartileRange; Range; MeanAbsoluteDeviation; RobustMeanAbsoluteDeviation; RootMeanSquared ; Skewness; Kurtosis; Variance; Uniformity |
| **GLCM (23)**  Autocorrelation; JointAverage; ClusterProminence; ClusterShade; ClusterTendency; Contrast; Correlation；DifferenceAverage; DifferenceEntropy; DifferenceVariance; JointEnergy; JointEntropy; Imc1; Imc2; Id; Idn; Idm; Idmn; InverseVariance; MaximumProbability; SumAverage; SumEntropy; SumSquares |
| **GLRLM (12)**  ShortRunEmphasis; LongRunEmphasis; GrayLevelNonUniformity; RunLengthNonUniformity; RunLengthNonUniformityNormalized; RunPercentage; LowGrayLevelRunEmphasis; HighGrayLevelRunEmphasis; ShortRunLowGrayLevelEmphasis; ShortRunHighGrayLevelEmphasis; LongRunLowGrayLevelEmphasis; LongRunHighGrayLevelEmphasis |
| **GLSZM (12)**  SmallAreaEmphasis; LargeAreaEmphasis; GrayLevelNonUniformity; SizeZoneNonUniformity ; SizeZoneNonUniformityNormalized; ZonePercentage; LowGrayLevelZoneEmphasis; HighGrayLevelZoneEmphasis; SmallAreaLowGrayLevelEmphasis; SmallAreaHighGrayLevelEmphasis; LargeAreaLowGrayLevelEmphasis; LargeAreaHighGrayLevelEmphasis |
| **GLDM (11)**  SmallDependenceEmphasis; LargeDependenceEmphasis; GrayLevelNonUniformity; DependenceNonUniformity; DependenceNonUniformityNormalized; DependenceEntropy; DependenceVariance; SmallDependenceLowGrayLevelEmphasis; SmallDependenceHighGrayLevelEmphasis; LargeDependenceLowGrayLevelEmphasis; LargeDependenceHighGrayLevelEmphasis |
| **NGTDM (5)**  Coarseness; Contrast; Busyness; Complexity; Strength |

**Table S5.** The performance of different models in the training cohort.

| Model | [Machine Learning](https://blog.csdn.net/hustlei/article/details/121803226) |  | Training cohort (182) | | | | Hyperparameters |
| --- | --- | --- | --- | --- | --- | --- | --- |
|  |  | Cutoff value | AUC (95% CI) | Specificity (%) | Sensitivity (%) | Accuracy (%) |  |
| Model 1: Clinical | SVM | 0.57 | 0.73 (0.66, 0.80) | 80.25 (71.58, 88.92) | 58.42 (48.80, 68.03) | 68.13 (61.36, 74.90) | C=0.9; gamma=0.1; kernel='linear'; shrinking=True; tol=0.001; |
|  | RF | 0.56 | 0.71 (0.63, 0.78) | 75.31 (65.92, 84.70) | 55.45 (45.75, 65.14) | 64.29 (57.32, 71.25) | max_depth=2; max_features=0.8; max_leaf_nodes=None; max_samples=0.4; min_samples_leaf=5; min_samples_split=25; n_estimators=50; |
|  | Bagging Decision Tree | 0.56 | 0.73 (0.64, 0.78) | 44.44 (33.62, 55.27) | 84.16 (77.04, 91.28) | 66.48 (59.63, 73.34) | estimator_max_depth=2; estimator_min_samples_split=25; max_features=0.2; max_samples=0.35; n_estimators=60; |
|  | XGBoost | 0.54 | 0.73 (0.70, 0.80) | 79.01 (70.14, 87.88) | 55.45 (45.75, 65.14) | 65.93 (59.05, 72.82) | colsample_bytree=0.5; eta=0.1; gamma=0.5; learning_rate=0.1; max_depth=2; n_estimators=50; scale_pos_weight=0.8; subsample=0.4; |
|  | LR | 0.51 | 0.71 (0.64, 0.79) | 77.78 (68.72, 86.83) | 56.44 (46.77, 66.11) | 65.93 (59.05, 72.82) | C=0.01; max_iter=1000; penalty='l2'; solver='liblinear'; tol=0.001; |
| Model 2: PVAT | SVM | 0.55 | 0.75 (0.68, 0.82) | 80.25 (71.58, 88.92) | 54.46 (44.74, 64.17) | 65.93 (59.05, 72.82) | C=0.9; gamma=0.1; kernel='poly'; |
|  | RF | 0.58 | 0.75 (0.61, 0.82) | 69.14 (59.08, 79.20) | 67.33 (58.18, 76.47) | 68.13 (61.36, 74.90) | max_depth=2; max_features=0.7; max_leaf_nodes=None; max_samples=0.5; min_samples_leaf=15; min_samples_split=25; n_estimators=80; |
|  | Bagging Decision Tree | 0.60 | 0.80 (0.74, 0.86) | 87.65 (80.49, 94.82) | 53.47 (43.74, 63.19) | 68.68 (61.94, 75.42) | max_depth=2; max_features=0.5; max_samples=0.7; min_samples_split=25; min_samples_leaf=15; n_estimators=80; |
|  | XGBoost | 0.51 | 0.79 (0.72, 0.85) | 70.37 (60.43, 80.31) | 69.31 (60.31, 78.30) | 69.78 (63.11, 76.45) | colsample_bytree=0.4; learning_rate=0.01; max_depth=2; n_estimators=100; scale_pos_weight=0.8; subsample=0.5; |
|  | LR | 0.43 | 0.71 (0.63, 0.78) | 58.02 (47.28, 68.77) | 79.21 (71.29, 87.12) | 69.78 (63.11, 76.45) | C=0.01; max_iter=1000; solver='liblinear'; tol=0.01; |
| Model 3: Plaque | SVM | 0.53 | 0.76 (0.69, 0.82) | 65.43 (55.07, 75.79) | 77.23 (69.05, 85.41) | 71.98 (65.45, 78.50) | C=0.05; gamma=0.01; kernel='linear'; ; |
|  | RF | 0.55 | 0.79 (0.72, 0.85) | 66.67 (56.40, 76.93) | 69.31 (60.31, 78.30) | 68.13 (61.36, 74.90) | max_depth=2; max_features=0.68; max_samples=0.85; min_samples_split=25; min_samples_leaf=15; n_estimators=80; |
|  | Bagging Decision Tree | 0.55 | 0.81 (0.75, 0.87) | 70.37 (60.43, 80.31) | 80.20 (72.43, 87.97) | 75.82 (69.60, 82.04) | max_depth=2; min_samples_split=35; max_features=0.45; max_samples=0.4; n_estimators=80; |
|  | XGBoost | 0.57 | 0.82 (0.75, 0.88) | 77.78 (68.72, 86.83) | 64.36 (55.02, 73.70) | 70.33 (63.69, 76.97) | colsample_bytree=0.6; gamma=0.3; learning_rate=0.01; max_depth=2; n_estimators=80; scale_pos_weight=1; subsample=0.25; |
|  | LR | 0.49 | 0.77 (0.70, 0.83) | 69.14 (59.08, 79.20) | 74.26 (65.73, 82.78) | 71.98 (65.45, 78.50) | C=0.05; max_iter=1000; solver='liblinear'; tol=0.01; |
| Model 4: PVAT+Plaque | SVM | 0.52 | 0.81 (0.74, 0.87) | 69.14 (59.08, 79.20) | 80.20 (72.43, 87.97) | 75.27 (69.01, 81.54) | C=0.5; gamma=0.1; kernel='linear'; ; |
|  | RF | 0.58 | 0.87 (0.81, 0.92) | 51.85 (40.97, 62.73) | 93.07 (88.12, 98.02) | 74.73 (68.41, 81.06) | max_depth=2; max_features=0.45; max_samples=0.9; min_samples_split=20; n_estimators=50; bootstrap=True; |
|  | Bagging Decision Tree | 0.57 | 0.86 (0.81, 0.91) | 83.95 (75.96, 91.94) | 73.27 (64.64, 81.90) | 78.02 (72.01, 84.04) | max_depth=2; min_samples_split=35; max_features=0.55; max_samples=0.55; n_estimators=90; |
|  | XGBoost | 0.50 | 0.87 (0.81, 0.91) | 83.95 (75.96, 91.94) | 70.30 (61.39, 79.21) | 76.37 (70.20, 82.55) | colsample_bytree=0.3; gamma=0.5; learning_rate=0.001; max_depth=2; n_estimators=80; min_child_weight=4; scale_pos_weight=1; subsample=0.52; |
|  | LR | 0.47 | 0.81 (0.74, 0.87) | 67.90 (57.73, 78.07) | 81.19 (73.57, 88.81) | 75.27 (69.01, 81.54) | C=0.05; max_iter=1000; solver='liblinear'; tol=0.01; |
| Model 5: Combined | SVM | 0.56 | 0.85 (0.79, 0.90) | 79.01 (70.14, 87.88) | 77.23 (69.05, 85.41) | 78.02 (72.01, 84.04) | C=0.05; gamma=0.1; kernel='linear'; ; |
|  | RF | 0.56 | 0.87 (0.81, 0.92) | 79.01 (70.14, 87.88) | 76.24 (67.94, 84.54) | 77.47 (71.40, 83.54) | max_depth=2; max_features=0.15; max_samples=0.85; min_samples_leaf=15; min_samples_split=25; n_estimators=100; bootstrap=True; |
|  | Bagging Decision Tree | 0.54 | 0.87 (0.82, 0.92) | 79.01 (70.14, 87.88) | 81.19 (73.57, 88.81) | 80.22 (74.43, 86.00) | max_depth=2; min_samples_split=25; max_features=0.4; max_samples=0.35; n_estimators=60; |
|  | XGBoost | 0.50 | 0.87 (0.83, 0.92) | 77.78 (68.72, 86.83) | 78.22 (70.17, 86.27) | 78.02 (72.01, 84.04) | colsample_bytree=0.4; eta=0.1; gamma=0.9; learning_rate=0.001; max_depth=2; n_estimators=100; scale_pos_weight=0.8; subsample=0.4; |
|  | LR | 0.49 | 0.85 (0.79, 0.90) | 76.54 (67.32, 85.77) | 74.26 (65.73, 82.78) | 75.27 (69.01, 81.54) | C=0.05; max_iter=1000; solver='liblinear'; tol=0.01; |

Note. AUC = area under the curve; PVAT, perivascular adipose tissue; SVM, support vector machines; RF, random forests; XGBoost, extreme gradient boosting; LR, logistic regression.

**Table S6.** Summary of Studies on Atherosclerosis Imaging and Risk Assessment

| **Study / Document** | **Core Objective** | **Key Imaging Feature Analysis** | **Comparison Benchmark/Group** | **Main Findings** | **Inclusion/Comparison of Traditional Quantitative Metrics (Volume, Calcification, Low-Attenuation Volume)** |
| --- | --- | --- | --- | --- | --- |
| Ours | Detecting symptomatic carotid atherosclerosis using machine learning (ML) combining plaque and PVAT radiomics. | Carotid plaque radiomic features, Perivascular Adipose Tissue (PVAT) radiomic features, clinical data (e.g., cholesterol). | Clinical model, plaque-only radiomics model, PVAT-only radiomics model, combined model. | Combined plaque+PVAT radiomics model significantly outperformed clinical model (AUC 0.86 vs 0.67) for identifying symptomatic patients. SHAP analysis highlighted plaque texture and cholesterol as key predictors. | No (Comparison focused on clinical vs. different radiomic model combinations, not directly against models based solely on traditional metrics). |
| 2025 Cui Z, et al. [2] | Evaluating ischemic stroke severity in symptomatic patients using ML based on carotid plaque and white matter lesion characteristics. | Stenosis degree, presence of intraplaque hemorrhage (IPH), plaque component volumes (lipid, fibrous, calcification, IPH), white matter lesion burden. | Stenosis degree, IPH presence, traditional logistic regression models. | ML models integrating plaque component volumes, white matter lesions, and other features achieved better predictive performance for stroke severity than models based on single features or traditional regression. | Yes (Used as input features), but the primary comparison was between different modeling approaches, not the fundamental value of traditional metrics alone. |
| 2022 Trandafir C, et al. [3] | Assessing the association between low-density plaque areas on CTA and symptomatic carotid arteries. | Stenosis degree, low-density plaque area (<25 HU or corrected minimum plaque CT value). | Asymptomatic carotid arteries. | Low-density areas were more prevalent in symptomatic arteries, potentially independent of stenosis degree. | Yes (Focused on the low-density feature), but centered on this single traditional metric without comparison to newer techniques or comprehensive volume analysis. |
| 2018 Goeller M, et al. [4] | Evaluating the relationship between coronary PVAT attenuation and high-risk plaque characteristics in ACS vs. stable CAD. | Coronary Perivascular Adipose Tissue (PVAT) CT attenuation. | Patients with stable coronary artery disease (CAD) vs. Acute Coronary Syndrome (ACS). | Higher PVAT attenuation was associated with high-risk plaque characteristics in patients with ACS compared to those with stable CAD. | No (Focus on PVAT characteristics; provides context for PVAT research). |
| 2018 Oikonomou EK, et al. [5] | Establishing the Fat Attenuation Index (FAI) derived from coronary PVAT CT attenuation as a novel biomarker for cardiovascular risk. | Coronary PVAT CT attenuation (measured as Fat Attenuation Index - FAI). | Traditional risk factors/scores, cardiovascular events (outcomes). | FAI improved cardiovascular risk prediction independently of traditional risk factors and coronary artery calcium score. | No (Focus on PVAT/FAI; provides context for PVAT research). |
| 2021 Narula J, et al. [6] | Providing expert consensus on the non-stenosis aspects of coronary CTA plaque assessment for risk stratification. | Plaque morphology, composition (qualitative/quantitative), burden, high-risk features (low attenuation, positive remodeling, spotty calcification, napkin-ring sign). | N/A (Consensus document, not a comparative study). | Systematically describes the assessment of plaque morphology, composition, burden, and high-risk features for risk stratification, emphasizing the role of these established (traditional) metrics. | Yes (Defines and emphasizes the importance and assessment methods for these traditional quantitative and qualitative metrics). |

Note. ML, machine learning; PVAT, perivascular adipose tissue; IPH, intraplaque hemorrhage; CAD, coronary artery disease; ACS, Acute Coronary Syndrome; FAI, Fat Attenuation Index.

**
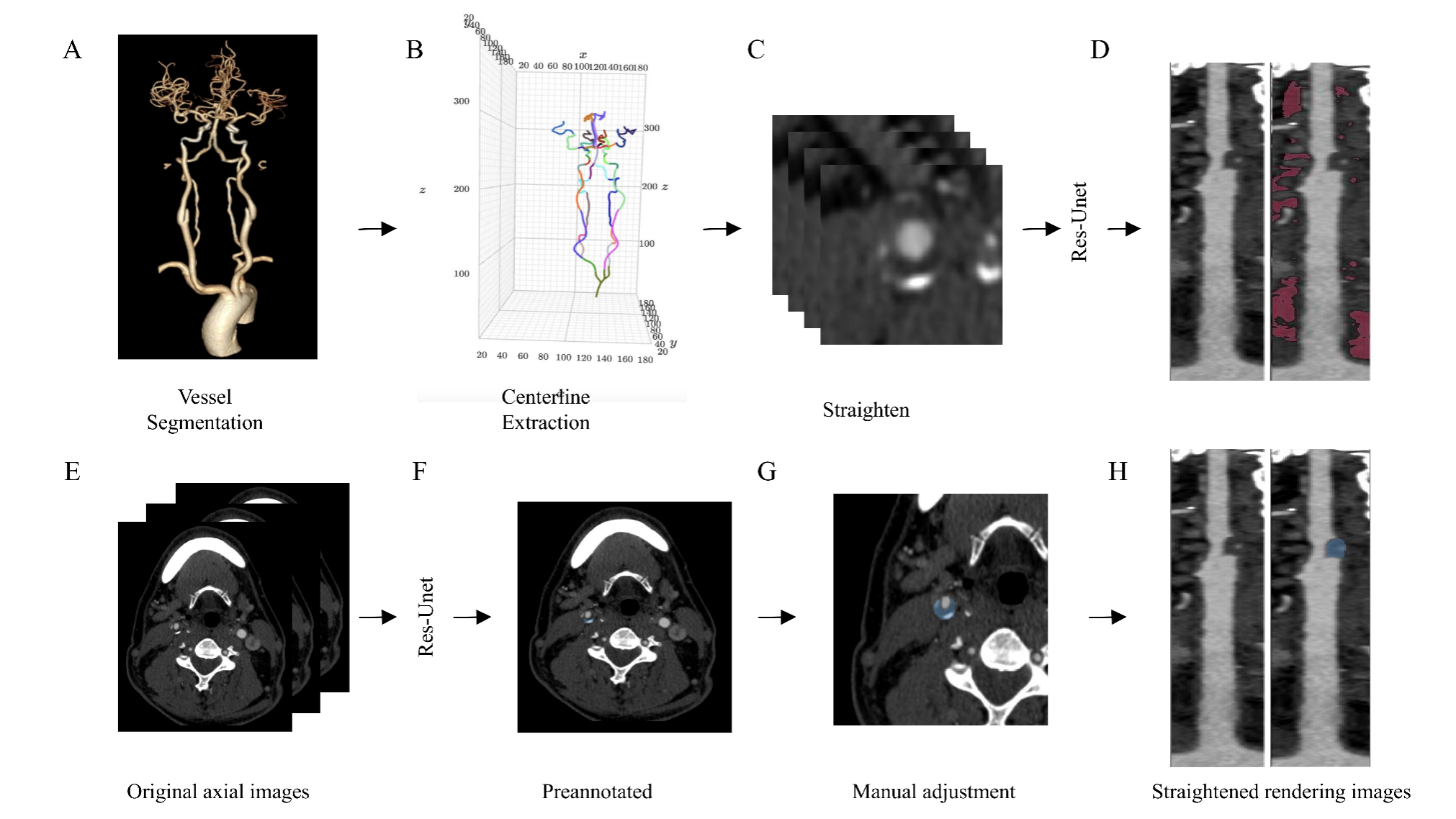
**

**Fig. S1 Segmentation Process for Pericarotid Adipose Tissue (PVAT) (A-D) and Plaque (E-H):** PVAT segmentation process: (A-B) Vascular structures were segmented from the CTA images using post-processing, and the vessel centerline was extracted. Specific topological structures were used as prior knowledge to ensure segmentation accuracy. (C-D) A straightened rendering image of the carotid artery was generated using the extracted centerline, and the ResU-Net model was applied to segment the vessel lumen in the straightened rendering image. PVAT was defined as the adipose tissue located at a radial distance from the vessel wall equal to the adjacent vessel lumen diameter. The region was selected based on an attenuation value range of -190 HU to -30 HU. Plaque segmentation process: (E-F) Three-dimensional ResU-Net was applied to the original axial images for initial plaque segmentation, generating preliminary annotations. (G-H) manual adjustments were made to the preliminary annotations to ensure segmentation accuracy. Finally, straightened rendered images were generated from the adjusted segmented plaques.

**
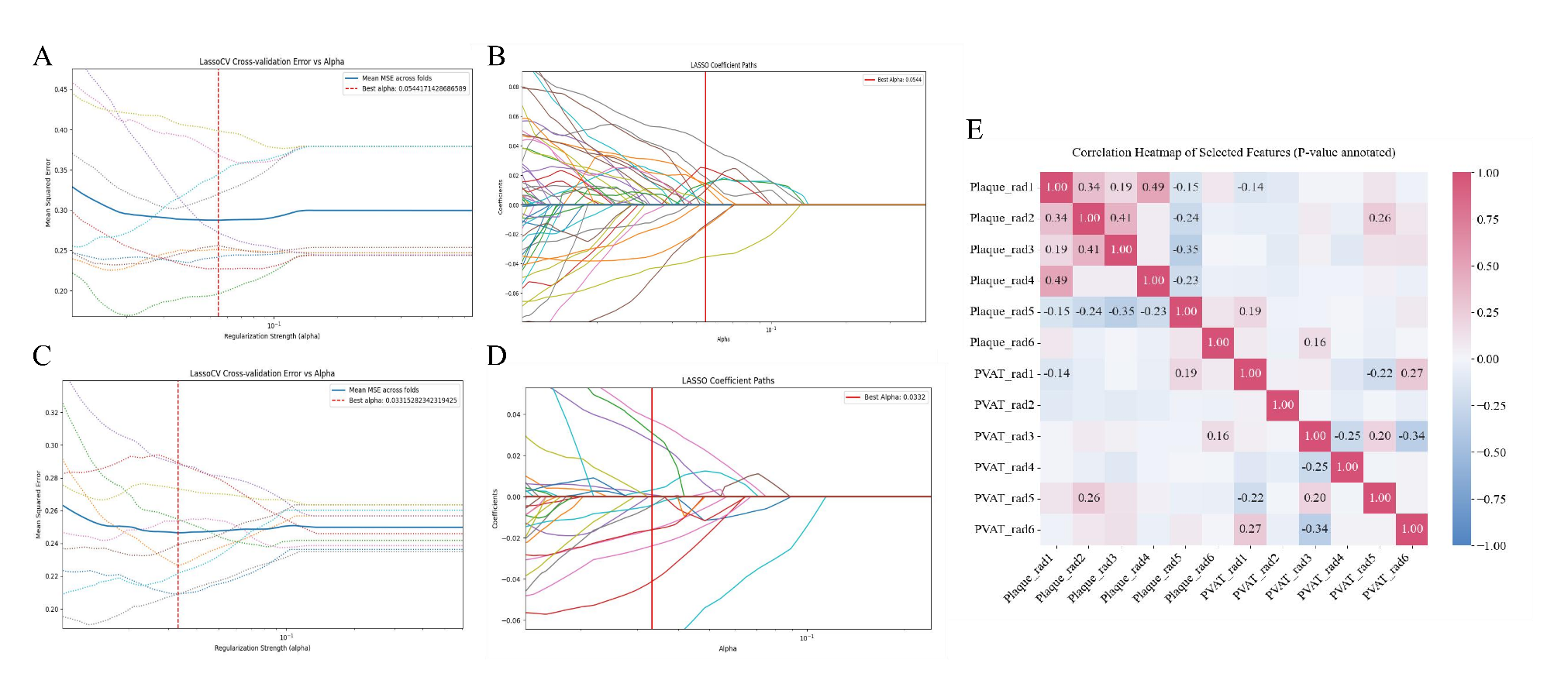
**

**Fig. S2 (A-B)** illustrates the process of radiomic feature selection for carotid plaque, including the LASSO coefficient path plot and the visualization of the selected regularization parameter. The blue line represents the mean squared error across all cross-validation folds, while the red vertical line indicates the optimal alpha value chosen. **(C-D)** describe the feature selection process for perivascular adipose tissue (PVAT) radiomics. **(G)** presents the correlation heatmap between PVAT and plaque radiomic features, with the correlation coefficients displayed numerically.

**References**

1. Luo W, Lv P, Zhang R, Qiu Q, Lin J. Additive value of perivascular fat density to CT angiography characteristics of carotid plaques in predicting symptomatic carotid plaques. Eur Radiol. 2025. doi:10.1007/s00330-025-11713-y.

2. Cui Z, Xu S, Miu J, Tang Y, Pan L, Cao X, et al. Development and Validation of a Fusion Model Based on Carotid Plaques and White Matter Lesion Burden Imaging Characteristics to Evaluate Ischemic Stroke Severity in Symptomatic Patients. J Magn Reson Imaging. 2025;61:648-660.

3. Trandafir C, Laurent-Chabalier S, Cosma C, Frandon J, Thouvenot E, Renard D. Association of symptomatic atherosclerotic carotid arteries with plaque areas showing low densities on computed tomographic angiography. Eur J Neurol. 2022;29:1056-1061.

4. Goeller M, Achenbach S, Cadet S, Kwan AC, Commandeur F, Slomka PJ, et al. Pericoronary Adipose Tissue Computed Tomography Attenuation and High-Risk Plaque Characteristics in Acute Coronary Syndrome Compared With Stable Coronary Artery Disease. JAMA Cardiol. 2018;3:858-863.

5. Oikonomou EK, Marwan M, Desai MY, Mancio J, Alashi A, Hutt Centeno E, et al. Non-invasive detection of coronary inflammation using computed tomography and prediction of residual cardiovascular risk (the CRISP CT study): a post-hoc analysis of prospective outcome data. Lancet. 2018;392:929-939.

6. Narula J, Chandrashekhar Y, Ahmadi A, et al (2021) SCCT 2021 Expert Consensus Document on Coronary Computed Tomographic Angiography: A Report of the Society of Cardiovascular Computed Tomography. J Cardiovasc Comput Tomogr DOI:10.1016/j.jcct.2020.11.001
